# Development and Psychometric Validation of the Cyber-Self Scale (CSS) in Saudi Arabia

**DOI:** 10.62641/aep.v53i1.1758

**Published:** 2025-01-05

**Authors:** Elsaeed A. Dardara, Khalid A. Al-Makhalid

**Affiliations:** ^1^Psychology Department, Faculty of Arts, Minia University, 61519 Minia, Egypt; ^2^Psychology Department, College of Education, Umm Al-Qura University, 24381 Mecca, Saudi Arabia

**Keywords:** Cyber-Self, E-emotional, cyber relationship motives, cyber psychology

## Abstract

**Background:**

Measuring adolescents’ and youths’ perception of their Cyber-Self can enhance the understanding of how digital technology influences identity formation. While psychological literature offers numerous measures of the self, there is a notable lack of studies addressing the measurement of the Cyber-Self. This study aims to evaluate the reliability, factorial- and criterion-related validity, and measurement invariance of the Cyber-Self Scale (CSS) across age and gender among the youth and adolescents in Saudi Arabia.

**Methods:**

The Cyber Relationship Motives (CRM) and E-Emotional Questionnaire (EEQ) were administered to students at Umm Al-Qura University (*N* = 335), aged 17–31 years (39.7% male, 60.3% female; mean (M) = 21.75, standard deviation (SD) = 2.17).

**Results:**

The results indicated significant positive correlations between the sub-components of the CRM and EEQ. One item was selected based on two criteria: the highest correlation with other items and the highest correlation with the general factor. A total of 12 items were identified as the final form of the CSS, which demonstrated acceptable internal consistency for both male and female participants. Confirmatory factor analysis (CFA) revealed that the CSS model fit the data well, with all 12 items meeting the fit criteria for chi-square and root mean square error of approximation (RMSEA).

**Conclusion:**

The Arabic version of the CSS is sufficiently reliable and valid for use among Arabic-speaking adolescents and youth. Further research is recommended to examine its measurement invariance over extended periods.

## Introduction

Research in the field of cyberpsychology remains limited in both scope and number of studies. Current research, particularly studies employing robust methodologies, is still in its early stages [1]. Digitalization has transformed daily life, leading to greater integration and reliance on virtual environments—not only for social interaction but also for accessing essential services such as food, clothing, education, shopping, and electronic transactions. In an era of rapid digitalization, the distinction between the cyber and real worlds has become increasingly blurred [2]. The widespread use of cyberspace is no longer an exception; rather, it has evolved into a common, steadily growing phenomenon. Recent advancements, such as the metaverse and ChatGPT, exemplify the emergence of a new generation of digital technologies [3, 4].

The Internet offers a distinct platform for individuals to express alternative selves [5]. The concept of ‘Flow’ in online environments suggests that the ease of Internet use, combined with the enjoyment of the experience, can lead users to lose themselves in virtual reality [6]. Early research on the self in cyberspace focused on the theory of self-presentation, highlighting the Internet as an anonymous space where individuals interact through multiple selves [7]. This has given rise to what is often referred to as the ‘era of alternative self-expression’, driven largely by the use of virtual reality, particularly the metaverse. The metaverse seamlessly integrates virtual and real-world spaces, offering a multi-sensory, engaging, and adventurous experience for users [2]. Virtual reality, in particular, provides a unique space for the expression of alternative selves, especially for individuals with social anxiety who may struggle to express themselves in real life [5]. It allows such individuals to redefine their identities and adopt virtual personas that reflect their personalities and represent their egos in the real world [8, 9]. In this context, the Cyber-Self becomes an alternative self, utilized in virtual reality environments to enhance one’s identity through social networking platforms [10]. Furthermore, some argue that, in the metaverse, individuals can adopt unique, alternate identities represented by customizable avatars [11]. It can allow for modifications in appearance, body image, and facial expressions [12]. With the advent of digitalization, some researchers have proposed that the Cyber-Self constitutes a fourth dimension of the self alongside the real, ideal, and ought selves. This Cyber-Self is often referred to by various terms, including the digital self and the technical self [13]. The Cyber-Self interacts with both the actual self and others within cyberspace. Despite the increasing relevance of the Cyber-Self, studies that measure it—particularly concerning the motivational and emotional aspects of cyber interactions—remain limited. In contrast, numerous measures of the real self are well-established in psychological literature [14, 15]. Understanding and measuring the Cyber-Self is of particular importance in educational contexts, as virtual learning environments offer greater flexibility and wider accessibility at a lower cost [16, 17]. Students may identify with virtual environments or use them to escape the real self in favor of the Cyber-Self. Thus, the practical significance of this study lies in its potential to inform the development of guidance programs aimed at enhancing psychological well-being [18, 19].

### Cyber Relationship Motives

Researchers have explored the motivations driving individuals to form online friendships. Some researchers have hypothesized that these motivations include entertainment, social integration, relationship maintenance, meeting new people, and social compensation [20]. The present study identifies eight primary motives for engaging in cyber relationships: the need for adventure, the desire for invisibility, the inclination to meet new people, the ease of communication, curiosity, emotional support, the desire to escape from the real world, and the need for romantic relationships [21]. A study has also developed measures to assess the motives behind cyber relationships among university students, identifying key motivations such as identity concealment, meeting new people, ease of communication, curiosity, emotional support, social compensation, escape from reality, and romantic interests [22].

### E-Emotions

E-emotions refer to the emotional content experienced during interactions in cyberspace, commonly termed electronic emotions. These emotions arise from mutual exchanges between individuals who communicate via electronic devices [15]. The present study identifies four key components of E-emotions, as outlined by Zych *et al*. (2017) [15], in relation to the Cyber-Self: expressing E-emotions, perceiving E-emotions, facilitating the use of E-emotions, and managing E-emotions.

Cyber-psychological theory suggests that the self presented on social networking sites is not the real self. Individuals in the cybersphere may perceive and present themselves as more socially desirable, but they may not be able to fully achieve this [23]. According to virtual self-theory, the self in virtual reality becomes a virtual entity, and its representation is often inaccurate and prone to distortion [24]. Similarly, the Looking-Glass theory posits that the self is not innate but rather formed through social interaction, as individuals view themselves through the perspective of others. Adolescents, in particular, perceive cyberspace as a safer environment, enjoying the anonymity it offers [7]. The Cyber-Self, therefore, emerges as an alternate self, surfacing during interactions in cyberspace through social networking sites [25]. The current study addresses the existing gap in research on Cyber-Self measures. It examines the concept of the Cyber-Self, assuming the nature of its formation and identifying factors that contribute to its development. These factors are integrated into a general factor (G factor) based on three hypotheses:

- H1: Significant correlations exist between cyber relationship motives and E-emotions.
- H2: If cyber relationship motives and E-emotions form a general factor, this factor arises from the empirical observation that independent and diverse scales show positive correlations among cyber-motivations and E-emotions.
- H3: Cyber relationship motives and E-emotions converge into a single concept, referred to as the Cyber-Self.

## Materials and Methods

### Participants

The sample consisted of 335 students (60.3% female, 39.7% male) aged 17–31 (mean (M) = 21.75, standard deviation (SD) = 2.17) enrolled in applied and theoretical courses at Umm Al-Qura University, Saudi Arabia.

### Instruments 

#### Cyber Relationship Motives (CRM)

The CRM is a 24-item scale, rated on a five-point Likert scale ranging from 1 (strongly disagree) to 5 (strongly agree). It measures various motives for engaging in cyber relationships, including invisibility, the desire to meet new people, ease of communication, curiosity, emotional support, social compensation, escape into the virtual world, and romance. The scale demonstrates high reliability, with Cronbach’s alpha values ranging from 0.86 to 0.90. It also exhibits satisfactory internal consistency, with values ranging from 0.57 to 0.66 (*p* < 0.01) [22].

#### E-Emotional Questionnaire (EEQ)

Developed by Zych *et al*. (2017) [15], the EEQ measures the expression of feelings and emotions during cyberspace interactions. It contains 21 items, also rated on a five-point Likert scale from 1 (strongly disagree) to 5 (strongly agree). The EEQ has a Cronbach’s alpha reliability coefficient of 0.76 and shows strong internal consistency over time [15].

### Translation of CRM and EEQ to Arabic 

Instrument adaptation: The original English versions of the EEQ and CRM questionnaires were translated into Arabic using a systematic process to ensure accuracy and cultural appropriateness. First, the Arabic translations were created by the authors, who were bilingual in Arabic and English and familiar with cyber behaviour. These translations were then back-translated into English by two professional bilinguals who were blind to the original English versions to reduce bias and verify conceptual and linguistic equivalence between the Arabic and English versions. To further refine the translations, three bilingual experts specializing in the English language reviewed the Arabic versions. The translations were revised iteratively based on their feedback until all items were deemed satisfactory. Next, a professional English-Arabic translator, who had not seen the original questionnaires, translated the final Arabic versions back into English. The experts compared the original and back-translated English versions to ensure that conceptual and linguistic equivalence had been achieved. This process was repeated until full equivalence was confirmed. The final Arabic versions were pretested with a sample of 50 Arabic-speaking Saudi students to assess item clarity. Students were interviewed and asked to rate the clarity of the items. Based on their feedback, final adjustments were made to the Arabic version. For cultural adaptation, four items related to sexual experiences via social media were removed from the CRM, as they were deemed inappropriate for Saudi society. The remaining items were culturally neutral and suitable for application within this context.

### Data Collection Procedure

In this study, participation was voluntary, and all participants were required to be Internet users for at least 5–6 hours per day and to have active social media accounts. The participants completed the questionnaires in small groups of 10–15 students within the classroom setting after their lectures. Data collection took place between October 2020 and February 2021.

### Data Analysis

Qualitative variables were described using frequencies and percentages, while quantitative variables were characterized through measures of central tendency (mean, median), dispersion (standard deviation), and shape (skewness, kurtosis), as appropriate. The Kolmogorov‒Smirnov test, Q‒Q plots, and histograms were used to assess the normality of the data distribution. Pearson correlation coefficients were used to examine the associations and relationships between the subscales of the EEQ and CRM. Exploratory factor analysis (EFA) was performed to identify whether a general factor or group factors existed within the Cyber-Self Scale (CSS). Subsequently, confirmatory factor analysis (CFA) was conducted to evaluate the suitability of the extracted model for the general factor of the CSS. Model fit was assessed using chi-square, df, root mean square error of approximation (RMSEA), Standardized Root Mean Residual (SRMR), Comparative Fit Index (CFT), and Tucker-Lewis Index (TLI). In addition, a *t*-test was used to examine the ability of the CSS to distinguish between extreme groups, dividing the sample into two groups based on the upper and lower quartiles and comparing the means. To assess the internal consistency reliability of the CSS, Cronbach’s alpha, and the Guttman Spilt-Half coefficient were calculated. A *p*-value of <0.05 was considered indicative of statistical significance. The data analysis was performed using IBM SPSS 22 with AMOS 22 (SPSS for Windows ver.22.0; SPSS Inc., Chicago, IL, USA).

## Results

Table 1 shows Pearson’s correlation coefficients between the motives of cyber relationships and E-emotions, indicating that all correlations are positive and significant (*p* < 0.01). This provides evidence for the presence of a general factor, supporting H2.

**Table 1.** **The straight correlation coefficient of the EEQ and CRM**.

EEQ/CRM	Emotional expression	Emotional perception	Facilitating the use of emotions	Understanding and management of emotions
Invisibility	0.35^*^	0.29^*^	0.34^*^	0.32^*^
The desire to meet new people	0.30^*^	0.28^*^	0.31^*^	0.28^*^
Ease of communication	0.26^*^	0.32^*^	0.26^*^	0.22^*^
Curiosity	0.27^*^	0.29^*^	0.31^*^	0.26^*^
Emotional support	0.25^*^	0.25^*^	0.23^*^	0.28^*^
Social compensation	0.29^*^	0.25^*^	0.34^*^	0.23^*^
Resort to the virtual world	0.32^*^	0.35^*^	0.33^*^	0.24^*^
Romance	0.44^*^	0.33^*^	0.38^*^	0.28^*^

^*^ The correlation coefficients between subscales of the EEQ and CRM are positive and significant (*p* < 0.01). EEQ, E-Emotional Questionnaire; CRM, Cyber Relationship Motives.

To verify H2, we tested whether the sub-dimensions of the CRM and EEQ scales were related to a G factor using EFA with principal components analysis and the Kaiser criterion. The EFA extracted 25 factors in total—12 for boys and 13 for girls—with each factor having an Eigenvalue of 1. However, due to the difficulty in interpreting such many factors, we only included the first factor for boys and girls before rotation.

Table 2 shows several correlation coefficients deemed acceptable, with a threshold of 0.30 or higher. As a result, 40 out of 45 items (88.9%) were accepted for boys (*N* = 133), and 39 items (86.6%) were accepted for girls (*N* = 202). To verify H3, which posits that the EEQ and CRM can be reduced to a unified concept termed the Cyber-Self, only one item was selected from each subscale of the EEQ and CRM based on two criteria. The first criterion involved selecting the item with the highest correlation with all other items in the EEQ and CRM (see Table 2). The second criterion required choosing the item with the highest correlation with the first factor before rotation through EFA of the 45 items (see Table 3). This selection process resulted in a final set of 12 items that represent the CSS. As shown in Table 3, the 12 items showed high correlations with the EEQ and CRM, all of which were positively significant (*p* < 0.01).

**Table 2.** **The first factor before rotation for the CRM (items #1–24) and EEQ (items #25–45)**.

Item	Boys	Girls	Item	Boys	Girls	Item	Boys	Girls
1 CRM	0.42	0.45	16 CRM	0.41	0.40	31 EEQ	0.55	0.47
2 CRM	0.42	0.37	17 CRM	0.50	0.44	32 EEQ	0.53	0.48
3 CRM	0.48	0.49	18 CRM	0.45	0.20	33 EEQ	0.63	0.47
4 CRM	0.46	0.40	19 CRM	0.49	0.38	34 EEQ	0.62	0.21
5 CRM	0.38	0.57	20 CRM	0.49	0.47	35 EEQ	0.48	0.55
6 CRM	0.45	0.54	21 CRM	0.47	0.50	36 EEQ	0.58	0.51
7 CRM	0.31	0.42	22 CRM	0.39	0.47	37 EEQ	0.50	0.43
8 CRM	0.40	0.39	23 CRM	0.43	0.44	38 EEQ	0.48	0.44
9 CRM	0.15	0.41	24 CRM	0.42	0.39	39 EEQ	0.46	0.42
10 CRM	0.29	0.43	25 EEQ	0.44	0.55	40 EEQ	0.42	0.39
11 CRM	0.35	0.57	26 EEQ	0.53	0.54	41 EEQ	0.40	0.16
12 CRM	0.31	0.52	27 EEQ	0.78	0.47	42 EEQ	0.28	0.20
13 CRM	0.39	0.52	28 EEQ	0.56	0.46	43 EEQ	0.37	0.25
14 CRM	0.36	0.44	29 EEQ	0.50	0.43	44 EEQ	0.32	0.51
15 CRM	0.25	0.49	30 EEQ	0.48	0.54	45 EEQ	0.21	0.15

Significantly acceptable coefficients are 0.03 or greater (40 for boys, and 39 for girls).

**Table 3.** **CSS items with the highest correlation with EEQ and CRM (items #45)**.

Items			The highest correlation of the item with EEQ and CRM	
			Boys	Girls
1	CRM6	I can present the real me when I make friends online	0.51^*^	0.51^*^
2	CRM11	Because I can find friends who share my interests	0.52^*^	0.52^*^
3	CRM13	Because making friends online is easy	0.49^*^	0.46^*^
4	CRM17	Because making friends online is new for me	0.44^*^	0.56^*^
5	EEQ12	Because making friends online comforts my spirit	0.52^*^	0.53^*^
6	EEQ3	Because I cannot find friends in other places	0.57^*^	0.56^*^
7	CRM3	Because I want to forget my worries temporarily	0.52^*^	0.61^*^
8	CRM7	My friends use the Internet to make friends, so I want to try it	0.45^*^	0.59^*^
9	CRM20	I usually have emotions on Facebook, Tuenti, or Instagram	0.58^*^	0.48^*^
10	EEQ7	My contacts let me know through Facebook, Tuenti, or Instagram if they are happy or sad	0.55^*^	0.44^*^
11	CRM10	If I change the emotion expressed through Facebook, Tuenti, or Instagram I see new possibilities	0.56^*^	0.50^*^
12	EEQ14	I usually understand why a contact on Facebook, Tuenti, or Instagram feels sad or happy	0.48^*^	0.43^*^

^*^ The 12 items with the highest significant correlation (*p* < 0.01) of the scales CRM and EEQ. CSS, Cyber-Self Scale.

In Table 3, according to the first criterion for selecting CSS items, which is selecting the item with the highest correlation with the CRM and EEQ, Pearson’s correlation coefficients showed 12 items that were significantly related (*p* < 0.01).

The second criterion for selecting items to represent the Cyber-Self was based on the EFA results. Table 4 shows that, for the total sample, the item with the highest commonality with the first factor before rotation was chosen.

**Table 4.** **The exploratory factor analysis EFA for the Cyber-Self-items**.

Items factor			The highest correlations of the item with the first factor before rotation
1	CRM6	I can present the real me when I make friends online	0.59
2	CRM11	Because I can find friends who share my interests	0.57
3	CRM13	Because making friends online is easy	0.55
4	CRM17	Because making friends online is new for me	0.53
5	EEQ12	Because making friends online comforts my spirit	0.52
6	EEQ3	Because I cannot find friends in other places	0.50
7	CRM3	Because I want to forget my worries temporarily	0.50
8	CRM7	My friends use the Internet to make friends, so I want to try it	0.50
9	CRM20	I usually have emotions on Facebook, Tuenti, or Instagram	0.48
10	EEQ7	My contacts let me know through Facebook, Tuenti, or Instagram if they are happy or sad	0.45
11	CRM10	If I change the emotion expressed through Facebook, Tuenti, or Instagram I see new possibilities	0.43
12	EEQ14	I usually understand why a contact on Facebook, Tuenti, or Instagram feels sad or happy	0.42
Eigenvalue			3.12
Variance explained by factor			26.02%

The EFA of the first factor for CSS before rotation. EFA, exploratory factor analysis.

Table 4 presents the results of EFA, which demonstrates the underlying factorial structure (*p* < 0.001). These results indicate high commonality among the items. Additionally, the tests of sphericity confirm that the scale is suitable for further factorial analyses.

### Confirmatory Factor Analysis (CFA)

**Fig. 1.**
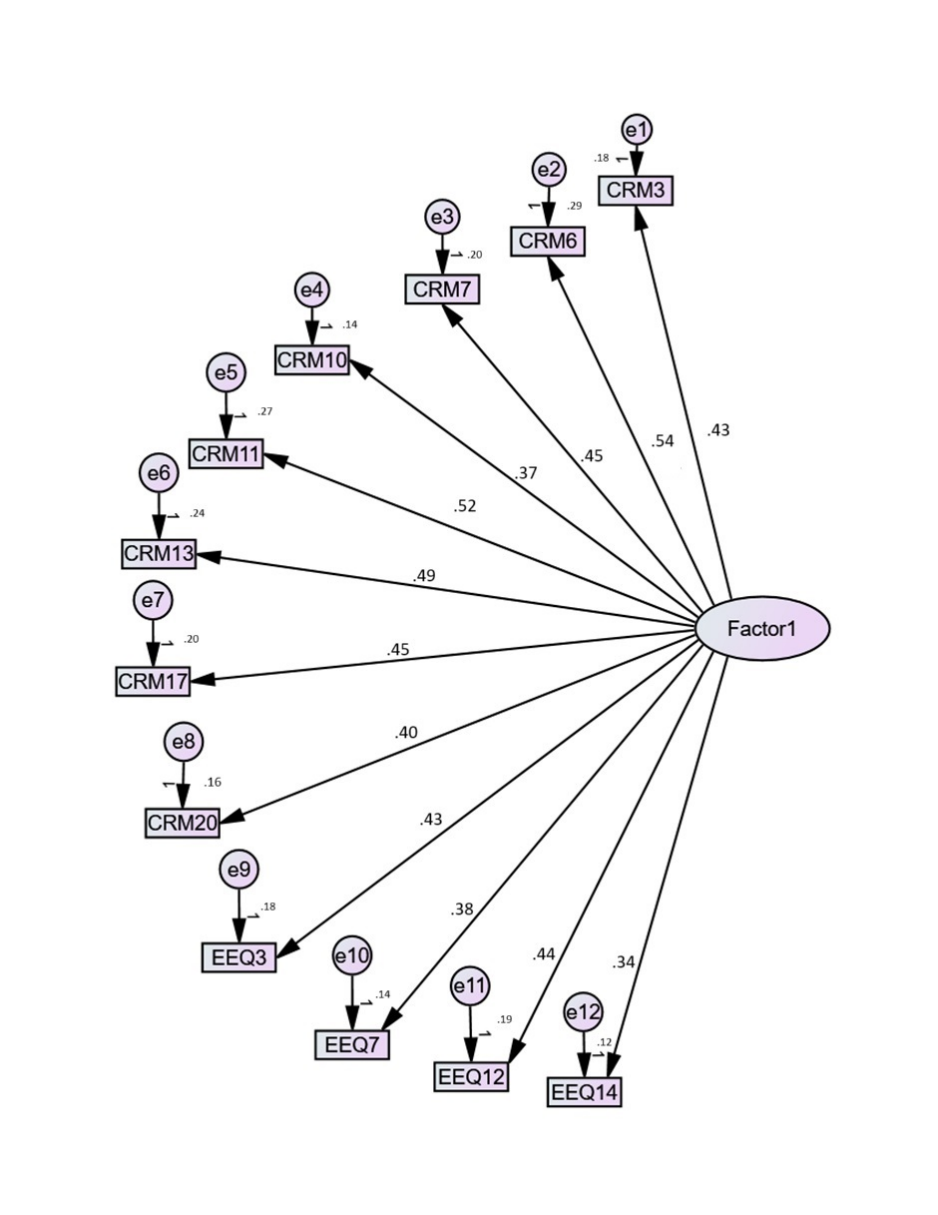
**Perceptual representation of the confirmatory factor analysis (CFA) model and factor loading (Through IBM SPSS 22 with AMOS 22, SPSS Inc., Chicago, IL, USA)**.

The 12-item CFA as a single-factor solution proved to be a valuable addition, particularly in situations where there is uncertainty regarding the best model solution for a specific culture, such as the Saudi sample. Fig. 1 illustrates the statistical analysis conducted using IBM SPSS 22 with AMOS 22, which confirmed the model’s adequacy through several goodness-of-fit indices. The CFA model yielded acceptable fit indices (chi-square = 165.576, df = 54 (*p* < 0.001), RMSEA = 0.082, SRMR = 0.048, CFI = 0.784, TLI = 0.734). The factor loadings from the CFA indicate that all 12 items demonstrate correlations close to or exceeding the CFA correlations, reflecting a good model fit (see Fig. 1).

The Convergent validity analysis of CSS items was supported. The CSS items were distinguished between contrasting groups using the quartiles of terminal comparison in the total sample (see Table 5). The results show a significant difference between contrasting groups.

**Table 5.** **Validity of discriminate of the CSS**.

CSS	Number of samples	Mean	Stander deviation	95% confidence interval of the difference		*t*-test	Degree of freedom	Sig.
				Lower	Upper			
	335	36.08	6.48	35.38	36.78	101.89	334	0.000

*t*-test value indicates discriminant validity of the CSS.

As shown in Table 5, the CSS’s discriminant validity was calculated between two extreme groups: the lower quartiles and the upper quartiles. The *t*-test showed a significant difference between the two groups to examine the CSS’s ability to discriminate.

Due to the novelty of the CSS in the Arab environment, finding an equivalent scale posed challenges. After discussions with professionals in psychology, the scales of Facebook and Twitter [26] were chosen as criteria for comparison. Correlation coefficients between the CSS, Facebook scale, and Twitter scale were calculated, yielding values of 0.90 and 0.92, respectively (*p* < 0.01).

Internal consistency reliability was assessed using Cronbach’s alpha, yielding coefficients of 0.83 for boys and 0.85 for girls. In addition, a split-half reliability analysis was conducted by dividing the items into two halves – one containing odd items and the other containing even items. The correlation coefficient between the two halves of the CSS items was 0.89, with a Guttman Spilt-Half Coefficient of 0.92, indicating a high level of reliability for the CSS.

## Discussion

The primary objective of this study was to develop an Arabic version of the CSS, derived from the CRM and EEQ. The results indicated a significantly positive relationship between the subscales of these measures, highlighting an overlap between the constructs they assess. While the study was correlational and does not establish causal relationships, the correlations suggest commonalities between the sub-dimensions of the CRM and EEQ, implying that these scales measure a unified behavioral characteristic. The findings supported H2, as a single factor extracted from the CRM and EEQ items was significantly related before rotation. This factor accounted for an acceptable amount of explained variance for both male and female participants, demonstrating that the sub-concepts of the CRM and EEQ are characterized by redundancy and overlap, as indicated by the strong correlations between items.

The 12 items related to this extracted factor described self-perception, motivations, and emotions during cyberspace interaction, aligning with the conceptualization of the Cyber-Self. This outcome is especially relevant given the limited research on emotional content in cyberspace [14, 15]. H3 suggested the possibility of creating a short form of the CSS. This was achieved by selecting one item from each sub-dimension of the CRM and EEQ based on two criteria: the highest item correlation with the overall scale and the highest correlation in the factor analysis before rotation. This process yielded 12 items that combined the shared characteristics of motives and emotions in cyberspace interactions, forming the final version of the CSS. The CSS addresses a gap in existing cyber behavior measures [15] and aligns with the modern psychological approach of breaking down phenomena into smaller components.

The study demonstrated that the CSS, derived from the CRM and EEQ, exhibits satisfactory reliability, with significant positive correlations between all 12 items and good internal consistency (Cronbach’s alpha). The CSS serves as a practical tool for screening students with a high Cyber-Self profile, particularly those who may exhibit signs of social withdrawal or increased immersion in cyberspace, potentially leading them to engage with harmful online content (e.g., pornography or violent and dark websites). The findings suggest that the Cyber-Self is a promising area of research in cyberpsychology, particularly given society’s increasing reliance on cyberculture and virtual applications such as the metaverse and artificial intelligence.

Objective measures, including physiological monitoring during virtual reality use, could enhance the effectiveness of virtual psychotherapy. In addition, the CSS offers valuable insights during therapy sessions with the youth and adolescents by helping therapists better understand and assess their cyber-related feelings and motivations, facilitating more accurate diagnosis and intervention. Future research should explore the development of new scales to measure cyber behaviour, cyber personality, and cyber well-being. Further studies may also investigate the relationship between the Cyber-Self and personality traits, as well as online behaviours such as social media usage, cyberbullying, and online dating activities.

Several limitations should be noted. First, the data in the current study were collected at a single point in time. Future studies should test equivalence across different time frames among populations from other countries. Second, the 12-item Arabic version of the CSS has not been tested. Future studies should examine whether this scale suffers from a method effect with participants from a different culture. Third, the study will need to develop a comprehensive model of the Cyber-Self. Given the novelty of the concept, there has been limited progress in advancing its theoretical and empirical understanding. To accelerate this progress, researchers in the field of Cyber-Self studies should coordinate their efforts, ideally conducting cross-cultural research, to establish a consensus on the construct more efficiently.

## Conclusion 

The present study highlights the need for further attention to measure the Cyber-Self. However, no definitive solution was reached using the CRM and EEQ scales, the study will need to be revisited once a comprehensive model of the Cyber-Self is developed. Given the novelty of the concept, there has been limited progress in advancing its theoretical and empirical understanding. To accelerate this progress, researchers in cyber-psychology should coordinate their efforts, ideally conducting cross-cultural research, to establish a consensus on the construct more efficiently. This collaboration is crucial, particularly as the integration of cyberspace into daily life continues to increase, demanding a more immediate understanding of the Cyber-Self. Delays, such as those experienced in reaching an agreement on the factor structure of intelligence, should be avoided in this emerging field, given the increasingly pervasive nature of our digital life.

## Availability of Data and Materials

The data and materials during this study are available from the corresponding author Dardara, Ph.D. (dardarae@mu.edu.eg).
